# The Incidence of Nonaffective, Nonorganic Psychotic Disorders in Older People: A Population-based Cohort Study of 3 Million People in Sweden

**DOI:** 10.1093/schbul/sby147

**Published:** 2018-10-19

**Authors:** Jean Stafford, Robert Howard, Christina Dalman, James B Kirkbride

**Affiliations:** 1 Division of Psychiatry, University College London, London, UK; 2 Department of Public Health Sciences, Karolinska Institutet, Stockholm, Sweden

**Keywords:** schizophrenia, late-onset, old age psychiatry

## Abstract

**Background:**

There are limited data on the epidemiology of very late-onset schizophrenia-like psychosis (VLOSLP) and how this relates to potential risk factors including migration, sensory impairment, traumatic life events, and social isolation.

**Methods:**

We followed up a cohort of 3 007 378 people living in Sweden, born 1920–1949, from their 60th birthday (earliest: January 15, 1980) until December 30 2011, emigration, death, or first recorded diagnosis of nonaffective psychosis. We examined VLOSLP incidence by age, sex, region of origin, income, partner or child death, birth period, and sensory impairments.

**Results:**

We identified 14 977 cases and an overall incidence of 37.7 per 100 000 person-years at-risk (95% CI = 37.1–38.3), with evidence that rates increased more sharply with age for women (likelihood ratio test: χ^2^(6) = 31.56, *P* < .001). After adjustment for confounders, rates of VLOSLP were higher among migrants from Africa (hazard ratio [HR] = 2.0, 95% CI = 1.4–2.7), North America (HR = 1.4, 95% CI = 1.0–1.9, *P* = .04), Europe (HR = 1.3, 95% CI = 1.2–1.4), Russian-Baltic regions (HR = 1.6, 95% CI = 1.4–1.9), and Finland (HR = 1.6, 95% CI = 1.5–1.7). VLOSLP risk was highest for those in the lowest income quartile (HR = 3.1, 95% CI = 2.9–3.3). Rates were raised in those whose partner died 2 years before cohort exit (HR = 1.1, 95% CI = 1.0–1.3, *P* = .02) or whose child died in infancy (HR = 1.2, 95% CI = 1.0–1.4, *P* = .05), those without a partner (HR = 1.9, 95% CI = 1.8–1.9) or children (HR = 2.4, 95% CI = 2.3–2.5), and those whose child had a psychotic disorder (HR = 2.4, 95% CI = 2.2–2.6).

**Interpretation:**

We identified a substantial burden of psychosis incidence in old age, with a higher preponderance in women and most migrant groups. Life course exposure to environmental factors including markers of deprivation, isolation, and adversity were associated with VLOSLP risk.

## Introduction

Although symptoms of nonaffective psychotic disorders typically emerge during adolescence or early adulthood,^1^ it has long been recognized that a subset of people experience their first episode of psychosis in old age,^2,3^ defined in the context of very late-onset schizophrenia-like psychosis (VLOSLP) as after 60 years old.^3^ The symptomatology underlying VLOSLP appears to be similar to psychosis in younger adults, although fewer negative symptoms are present.^4^ Epidemiology has been less well characterized,^5^ and while there is consistent evidence that VLOSLPs are more common in women than men,^4,6^ whether rates vary by age, migration, or other potential social determinants of risk, such as traumatic life events or social isolation, remains largely unexplored.

Previous findings with respect to age have been mixed, showing both increased^7^ and decreased VLOSLP rates with advancing age.^8–10^ It is also unclear whether these patterns differ between men and women. In line with the literature on psychosis incidence in younger adults,^11,12^ several studies have also reported higher risk among migrants,^13,14^ but this literature remains sparse, particularly outside of the United Kingdom. Indeed, in general, the VLOSLP literature has predominantly consisted of small-scale, cross-sectional studies. Although these have led to the identification of several potential risk factors, including sensory impairments,^15^ social isolation,^16,17^ premorbid schizotypal traits,^16^ and traumatic life events,^18–21^ results have not been consistently replicated.^22^ Epidemiological investigation in large, population-based longitudinal studies is largely lacking, with limited exceptions.^10^ Older people have consistently been omitted from studies that have elucidated a robust set of risk indicators for psychotic disorders at younger ages.^23–26^

The principal aim of this study was to delineate the epidemiology of VLOSLP in a national, population-based cohort of people living in Sweden since 1920. We aimed to examine variation in incidence rates by potential risk factors for VLOSLP, hypothesizing that advanced age, female sex, migrant status, lower socioeconomic status (SES), family history of psychotic disorders, sensory impairment, gestational exposure to World War II (WWII) (which could confer risk, in utero, via nutritional deficiencies or maternal trauma), social isolation, and death of a partner or child would increase risk.

## Methods

### Study Design and Setting

In Sweden, all people granted residency are given a unique national identification number, allowing record linkage across national health and administrative registers. “Psychiatry Sweden” is a linkage of these national registers for the study of psychiatric disorders. Using this data, we established a longitudinal cohort of people born between 1920 and 1949, and living in, or who immigrated to, Sweden on or after their 60th birthday. Participants born before 1932 were enumerated and identified from the 1960 and 1965 censuses, whereas those born since 1932 have been followed prospectively through the registers. In our study, participants were followed from their 60th birthday (earliest: January 15, 1980) until the end of follow-up (December 30, 2011), emigration from Sweden, dementia diagnosis, death or psychotic disorder diagnosis, whichever came first. We excluded those who died before age 60 years (*N* = 291 568), emigrated from Sweden before age 60 without return (*N* = 202 325), or were diagnosed with dementia before diagnosis with a psychotic disorder (*N* = 2570) (supplementary table S1).

### Outcome

We identified all participants recorded in the Swedish National Patient Register diagnosed with nonaffective psychotic disorder according to the International Classification of Diseases, Revisions 8–10 (ICD-8, -9, -10) since 1980 (supplementary table S1). The Swedish National Patient Register contains records of approximately 70% of all psychiatric admissions in the healthcare system (excluding primary care) from 1970, 83% by 1973, 97% from 1974 to 1983, 80%–95% from 1984 to 1986, and has been close to complete since 1987.^27^ Recording of outpatient data began in 1997 and was close to complete from 2001.

### Covariates

Data on age, sex, birth period, and region of birth were obtained from the Swedish Register of the Total Population. We categorized region of birth as Sweden, Africa, Asia, North America, Europe, Finland, South America, Oceania, Middle East, Russia-Baltic, and “Other.” To investigate the possible role of gestational exposure to maternal stressors experienced during WWII (September 1, 1939–September 2, 1945), we categorized participants into the following birth periods, based on earliest likely gestational date (see supplementary methods): 1920–1924, 1925–1929, 1930–1933, 1934–August 1939, September 1939–May 1946 (WWII gestation), and June 1946–1949. We created a disposable income variable, grouped into quartiles, based on all cohort members with disposable income from all sources (employment, welfare receipts, savings, investments) at age 60 recorded in the same calendar year, using data from the Longitudinal Integration Database for Health Insurance and Labour Market Studies (LISA). Using administrative registers, the LISA collects information annually on work-related information such as income, employment, education, and insurance for the total Swedish population aged 16 years and older. We linked participants to their children via the Multigenerational Register to derive a measure of psychosis family history, based on whether their biological children had ever received a psychotic disorder diagnosis. We also linked this register to the Cause of Death Register to obtain data on death of a child (biological and adopted) before cohort exit, before child age 12 months or age 18 years. These exposures were grouped as follows: “Had children, no child death” (reference), “Had no children,” and “Death of at least one child.” We also created a variable on death of a partner in the 2 years preceding cohort exit, using Census data (before 1990) or the LISA database thereafter, linked to the Cause of Death Register (see supplementary methods). This variable was grouped as follows: “Had a partner, but did not experience partner death (reference),” “Death of one or more partners,” and “Had no partner.” We created binary hearing and visual impairment variables using diagnoses from the National Patient Register recorded before cohort exit (ICD codes: supplementary table S1).

### Missing Data

Missing data were limited to income. For income, we included data at age 55–59 for those with missing data at age 60 years, where possible (*N* = 21 325; 0.72%). We conducted complete-case analyses, dropping those with remaining missing data on income from analyses (1.7%) (supplementary table S2).

### Statistical Analysis

We used Cox proportional hazards regression to model survival in the context of time to VLOSLP diagnosis in relation to exposures of interest, reported using hazard ratios (HR) with 95% CIs. Initially, we examined univariable associations between each exposure and the outcome, recording overall fit of each model using Akaike’s Information Criterion (AIC), where low scores indicated better fit. Using a forward-fitting modeling strategy, we added variables with the lowest AIC scores to a multivariable model with age, sex, and their interaction included as a priori confounders. Model building was tested via likelihood ratio test (LRT). Age was modeled as a time-varying covariate using Lexis expansion to examine age at-risk during follow-up, which was grouped into 5-year age bands between ages 60 and 90 years and older. In sensitivity analyses, we excluded migrants diagnosed with a psychotic disorder within 2 years of immigration to Sweden to mitigate the possibility of including prevalent cases in our sample. In addition, we conducted a sensitivity analysis to examine any differences in results after excluding those diagnosed with dementia within 2 years of diagnosis with VLOSLP, given that these individuals may be considered to be experiencing the dementia prodrome. We also tested our final model for violation of the proportional hazards assumption. Analyses were conducted using STATA, version 13.

## Results

From 3 007 378 people contributing 39 764 686 person-years of follow-up time, we identified 14 977 cases diagnosed with VLOSLP during the follow-up period, corresponding to a crude incidence rate of 37.66 per 100 000 person-years at-risk (95% CI = 37.06–38.27). After excluding participants with missing income data (1.7%), 2 955 796 cohort members were retained, including 14 825 cases. Median age-at-first diagnosis of VLOSLP was 68 years for men (interquartile range [IQR] = 64–74) and 70 years for women (IQR = 65–77; Mann-Whitney *P* ≤ .001). Compared with the remainder of the population, people with VLOSLP were more likely to be women (60% vs 50%), from the lowest income quartile (39% vs 22%), have no children (33% vs 15%), have children with a psychotic disorder (5% vs 2%), have no partner in the 2 years before cohort exit (71% vs 44%), be born outside of Sweden (14% vs 11%), and be born in the youngest birth period (table 1; all *P*s ≤ .001).

**Table 1.** Participant Characteristics

	All participants born in Sweden between 1920 and 1949 (*N* = 2 955 796)		
	Population at-risk (*N* = 2 940 971, 99.5%) *N* (%)	Cases (*N* = 14 825, 0.5%), *N* (%)	χ^2^ test
Sex			
Men	1 460 201 (49.65)	5974 (40.30)	χ^2^(1) = 516.23, *P* ≤. 001
Women	1 480 770 (50.35)	8851 (59.70)	
Region of origin			
Sweden	2 606 243 (88.62)	12 539 (84.58)	χ^2^(10) = 318.59, *P* ≤ .001
Africa	5034 (0.17)	38 (0.26)	
Asia	17 518 (0.60)	82 (0.55)	
North America	5538 (0.19)	42 (0.28)	
Europe	160 493 (5.46)	1099 (7.41)	
South America	8074 (0.27)	42 (0.28)	
Oceania	225 (0.01)	1 (0.01)	
Other	46 (0.00)	0 (0.00)	
Middle East	10 063 (0.34)	34 (0.23)	
Russia-Baltic	13 344 (0.45)	139 (0.94)	
Finnish	114 393 (3.89)	809 (5.46)	
Birth period			
1920–1924	504 290 (17.15)	5027 (33.91)	χ^2^(5) = 5000.00, *P* ≤ .001
1925–1929	432 028 (14.69)	3128 (21.10)	
1930–1934	405 822 (13.80)	2370 (15.99)	
1934–August 1939	404 116 (13.74)	1723 (11.62)	
WWII–May 1946	752, 654 (25.59)	2022 (13.64)	
Post–WWII–1949	442, 061 (15.03)	555 (3.74)	
Disposable income at age 60			
Lowest quarter	646 200 (21.97)	5724 (38.61)	χ^2^(3) = 4600.00, *P* ≤ .001
Second quarter	639 058 (21.73)	4744 (32.00)	
Third quarter	818 099 (27.82)	2722 (18.36)	
Highest quarter	837 614 (28.48)	1635 (11.03)	
Child with a psychotic disorder			
Yes	69 142 (2.35)	773 (5.21)	χ^2^(1) = 523.61, *P* ≤ .001
No	2 871 829 (97.65)	14 052 (94.79)	
Death of child under 12 months			
No children died aged under 12 months	2 473 160 (84.09)	9771 (65.91)	χ^2^(2) = 4000.00, *P* ≤ .001
Had no children aged under 12 months	433 838 (14.75)	4937 (33.30)	
1 or more children died	33 973 (1.16)	117 (0.79)	
Death of child aged 12 months–18 years			
No children died aged 12 months–18 years	2 482 177 (84.40)	9786 (66.01)	χ^2^(2) = 4000.00, *P* ≤ .001
Had no children aged 12 months–18 years	433 838 (14.75)	4 937 (33.30)	
1 or more children died aged 12 months–18 years	24 956 (0.85)	102 (0.69)	
Partner death 2 years before date of exit			
Had partner, no partner died	1 564 799 (53.21)	3883 (26.19)	χ^2^(2) = 4400.00, *P* ≤ .001
Had no partner	1 303 523 (44.32)	10 569 (71.29)	
1 or more partners died	72 649 (2.47)	373 (2.52)	
Sensory impairment			
Visual impairment	745 076 (25.33)	2037 (13.74)	χ^2^(1) = 1000.00, *P* ≤ .001
No visual impairment	2 195 895 (74.67)	12 788 (86.26)	
Hearing impairment	180 543 (6.14)	500 (3.37)	χ^2^(1) = 196.30, *P* ≤ .001
No hearing impairment	2 760 428 (93.86)	14 325 (96.63)	

### Incidence by Age and Sex

**Fig. 1.**
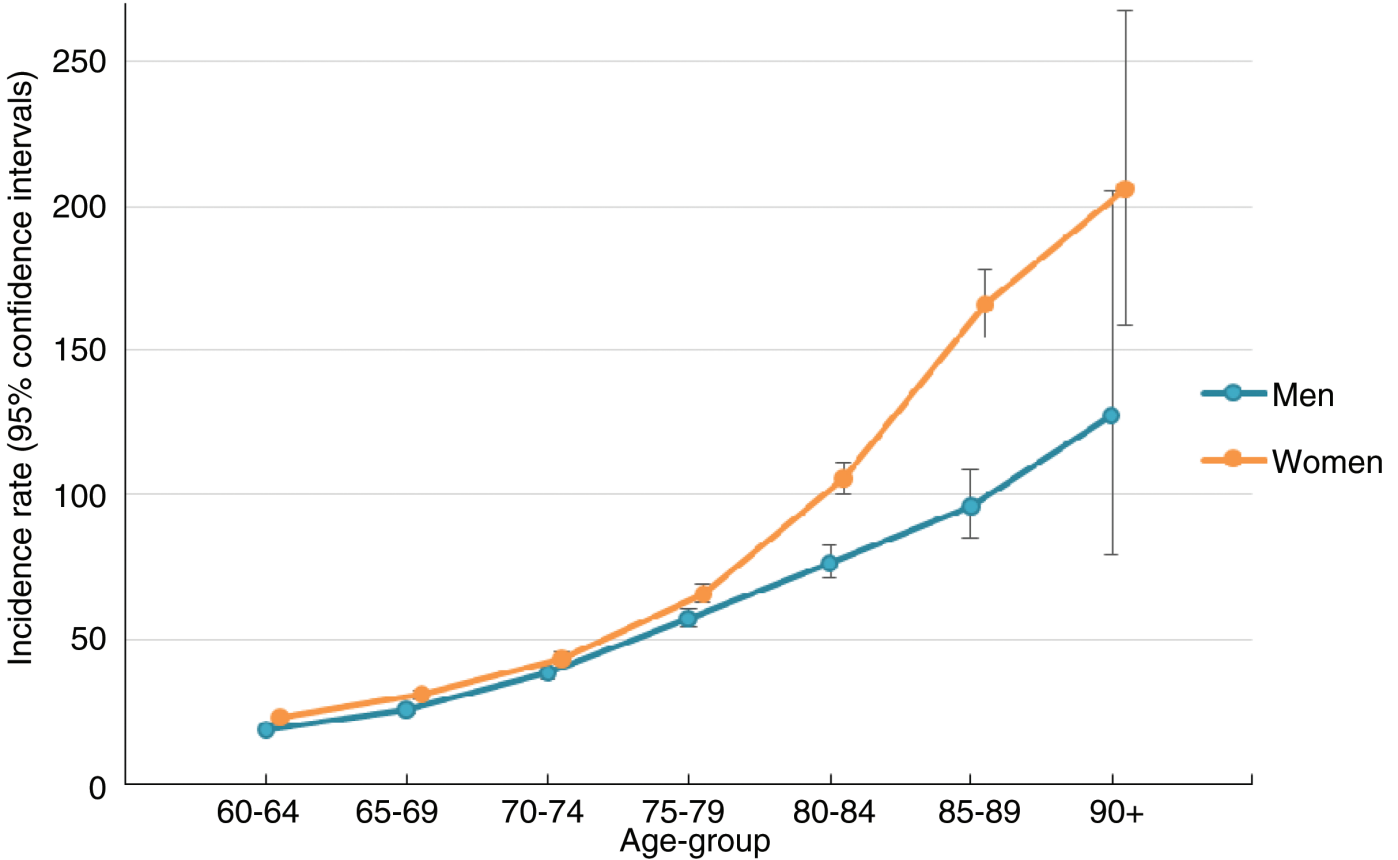
Crude incidence rates of very late-onset schizophrenia-like psychosis per 100 000 person-years at-risk by age and sex.

A significant interaction was observed between age and sex in crude and fully adjusted analyses (unadjusted model LRT: χ^2^(6) = 38.19, *P* < .001; adjusted model LRT: χ^2^(6) = 31.56, *P* < .001). This suggested that VLOSLP incidence increased with age for men and women, but at an accelerated rate for women after age 80 years (figure 1).

### Proportional Hazards Modeling

After adjustment for age and sex, we observed strong associations between almost all variables of interest and risk (hazard) of being diagnosed with VLOSLP (Adjustment 1, table 2). Full adjustment following multivariable model building led to some attenuation in observed associations (table 2), but most risk factors remained associated with VLOSLP. For example, migrants from Africa (HR = 1.98, 95% CI = 1.44–2.72), North America (HR = 1.38, 95% CI = 1.02–1.87), and Europe (HR = 1.32, 95% CI = 1.24–1.40) were at elevated VLOSLP risk after adjusting for all other covariates, including income at age 60. Those born in later birth periods, particularly those with gestational exposure to WWII (HR = 2.35, 95% CI = 2.20–2.50) or born after WWII (HR = 3.09, 95% CI = 2.79–3.42) were more likely to receive a diagnosis of VLOSLP compared with those born 1920–1924. Lower income at age 60 years was strongly associated with future risk of VLOSLP, with the highest rates in those in the lowest income quartile (HR = 3.07, 95% CI = 2.89–3.25). Participants whose children had a history of psychotic disorder were over twice as likely to develop VLOSLP than those without such a family history (HR = 2.40; 95% CI = 2.23–2.58), as were those without children (HR = 2.41, 95% CI = 2.32–2.50). Participants without a partner 2 years before cohort exit (HR = 1.86, 95% CI = 1.78–1.93) or whose partner died (HR = 1.14, 95% CI = 1.02–1.27) were also at higher VLOSLP risk. There was weak evidence that people who lost a child in infancy were more likely to develop VLOSLP than those with children who did not die in infancy (HR = 1.20, 95% CI = 1.00–1.44, *P* = .050), although death of a child before age 18 years was not associated with VLOSLP risk in our final model.

**Table 2.** Association Between Potential Risk Factors and Very Late-Onset Schizophrenia-like Psychosis Hazard Ratios (HR)

	Adjustment 1 HR (95% CI)^a^	Adjustment 2 HR (95% CI)^b^
Offspring with nonaffective psychotic disorder (NAPD) (ref: no offspring with NAPD)	2.04 (1.90–2.19)	2.40 (2.23–2.58)
Region of origin (ref: Sweden)		
Africa	3.51 (2.55–4.83)	1.98 (1.44–2.72)
Asia	1.93 (1.55–2.40)	1.01 (0.81–1.25)
North America	1.51 (1.12–2.05)	1.38 (1.02–1.87)
Europe	1.67 (1.56–1.77)	1.32 (1.24–1.40)
South America	2.19 (1.62–2.96)	1.11 (0.82–1.50)
Oceania	1.80 (0.25–12.76)	1.10 (0.16–7.83)
Middle East	1.11 (0.79–1.55)	0.69 (0.49–0.96)
Russia-Baltic	1.78 (1.50–2.10)	1.62 (1.37–1.91)
Finland	1.89 (1.76–2.03)	1.57 (1.46–1.69)
Birth period (ref: 1920–1924)		
1925–1929	0.92 (0.88–0.96)	1.03 (0.98–1.08)
1930–1934	1.08 (1.02–1.14)	1.34 (1.27–1.41)
1934–August 1939	1.29 (1.21–1.37)	1.63 (1.53–1.73)
September 1939–May 1946 (gestational exposure to WWII)	1.75 (1.65–1.87)	2.35 (2.20–2.50)
June 1946–1949	2.31 (2.08–2.55)	3.09 (2.79–3.42)
Disposable income at age 60 (ref: highest quartile (4))		
Income quartile 1 (lowest)	3.21 (3.03–3.41)	3.07 (2.89–3.25)
Income quartile 2	2.93 (2.76–3.10)	2.72 (2.56–2.88)
Income quartile 3	1.55 (1.46–1.65)	1.46 (1.37–1.55)
Death of child (ref: had children, none died):		
Had no children	2.97 (2.87–3.08)	2.41 (2.32–2.50)
1 or more children died aged under 12 months	1.31 (1.09–1.57)	1.20 (1.00–1.44)
1 or more children died aged 12 months–18 years	1.04 (0.86–1.27)	0.99 (0.81–1.20)
Death of partner 2 years before date of exit (ref: no partner died)		
Had no partner	2.22 (2.14–2.31)	1.86 (1.78–1.93)
1 or more partners died	1.15 (1.03–1.28)	1.14 (1.02–1.27)
Visual impairment (ref: no visual impairment)	0.22 (0.21–0.23)	0.24 (0.23–0.25)
Hearing impairment (ref: no hearing impairment)	0.45 (0.41–0.49)	0.55 (0.50–0.60)

^a^Adjustment 1: adjusted for age, sex, and their interaction.

^b^Adjustment 2: adjusted for age, sex, their interaction, and all exposures included in this table.

Interestingly, and contrary to our hypotheses, those with a history of sensory impairment were less likely to receive a diagnosis of VLOSLP (visual impairment HR = 0.24, 95% CI = 0.23–0.25, hearing impairment HR = 0.55, 95% CI = 0.50–0.60); this finding was independently present in the domains of visual impairment and hearing loss.

### Sensitivity Analyses

Results from the sensitivity analysis, excluding migrants who presented for psychosis within 2 years of arrival to Sweden, and who may have been prevalent cases, did not substantially differ from results based on the full sample (supplementary table S3). Results from an additional sensitivity analysis excluding those diagnosed with dementia within 2 years of diagnosis with VLOSLP were also very similar to results involving the full sample (supplementary table S4). 

### Proportional Hazards

There was some evidence that the proportional hazard assumption was violated for several variables (supplementary table S5). Inspection of the data, stratified by time (supplementary table S6), suggested the effects of income and sensory impairments on VLOSLP risk weakened over time (ie, in the youngest cohort), whereas death of a partner was only associated with risk in the youngest cohort.

## Discussion

### Summary of Findings

In this nationwide cohort study investigating the epidemiology of VLOSLP, we found substantial incidence after age 60 years. The overall incidence rate of 37.66 per 100 000 person-years at-risk (95% CI = 37.06–38.27) was toward the higher end of previously reported rates of VLOSLP. In a recent systematic review,^5^ the overall rate of nonaffective psychotic disorders in those aged 60 years and older was found to vary substantially across studies, ranging from 14.3 per 100 kpy in Northumberland (95% CI = 10.5–18.1)^28^ to 39.9 per 100 kpy in Camberwell (95% CI = 31.1–51.3).^14^ Rates increased with age beyond 80 years old and were generally higher in women than men; a disparity that widened with increased age. Rates were higher for those born later, including those with some gestational exposure to WWII. Consistent with epidemiological research in younger adult-onset samples, we found raised rates among some migrant groups, particularly from Africa and Europe. These findings were unlikely to be explained by prevalent cases among migrants, or by income, itself a strong predictor of VLOSLP. Unexpectedly, rates were lower in those with sensory impairments. Finally, we found higher rates among those without a partner or children, those whose children had a history of psychotic disorder, and those who had experienced the recent death of a partner or child in infancy.

### Strengths and Limitations

This is the largest population-based cohort study to examine the incidence of VLOSLP. We used Swedish registry data, which are highly complete and reliable for research purposes.^29,30^ This enabled us to include a relatively high number of cases and to obtain precise estimates for potential risk factors.

We note several study limitations, including the need to consider whether reliance on register-based diagnoses could have biased results. On the one hand, we may have underestimated true incidence; as those with VLOSLP may be less likely to contact services due to higher levels of functioning,^16^ and limited social contact.^31^ By contrast, given that recording of psychiatric diagnoses in Swedish registers only began in 1973, we may have included some prevalent cases, which would have overestimated incidence. Nonetheless, our follow-up period began in 1980 and we excluded those with a recorded psychotic disorder in the 7 years prior. Register coverage improved over this washout period, during which most prevalent cases would be expected to present to services. We may have expected more prevalent cases among older birth cohorts, who were more likely to have experienced the major risk period for psychosis (late teens through to early 30s^32^) before routine registration of psychiatric diagnoses began. By contrast, we found stronger VLOSLP risk in our younger birth cohorts. This may be explained by differential ascertainment bias over time, whereby younger cohorts were more likely to be diagnosed with VLOSLP after 60 years old, either because of more complete register coverage since 1987 or because of improved clinical awareness of VLOSLP as a distinct set of syndromes from dementias in later life.

To mitigate the possibility of VLOSLP representing misclassified dementia with psychosis symptoms, we excluded those diagnosed with dementia before psychotic disorder (*N* = 2570). In general, when an older patient presents to services with psychotic symptoms, we would expect dementia to be assessed and ruled out before a psychotic disorder diagnosis was given. However, we cannot exclude the possibility of psychosis representing misdiagnosed dementia in some cases, or the reverse. It is also possible that, in some patients, VLOSLP represents a prodrome for future dementia.^33^ Correspondingly, a Danish register-based study identified higher rates of subsequent dementia in those with VLOSLP compared with the general population and osteoarthritis patients.^34^ To examine whether our findings were influenced by including those who may be experiencing the dementia prodrome, we conducted a sensitivity analysis excluding individuals diagnosed with dementia in the 2 years following diagnosis with VLOSLP. Results were very similar to those involving the full sample, suggesting that our findings are unlikely to be explained by the inclusion of this group.

In this study, we could not link our cohort with their parents; hence we could not delineate second-generation migrants from the Swedish-born population. However, we would not expect a large number of second-generation migrants in this cohort given the birth periods covered. We were also, therefore, unable to investigate parental history of psychotic disorder; instead we used offspring psychotic illness as an indirect proxy. This will have overestimated the prevalence of psychosis family history, and our strong estimates for this variable may therefore be conservative. We also had to make some assumptions about coding death of a partner in the 2 years before cohort exit (supplementary methods). We do not consider this will have introduced any substantial biases in our data. In addition, we did not examine other mental health diagnoses such as depression, substance abuse, or bipolar disorder in this study. Future studies examining premorbid mental health conditions in those with VLOSLP could provide valuable insights into the mental health trajectories of this group throughout adult life, before the emergence of late-life psychosis. Finally, the proportional hazards assumption was violated for several exposures, warranting further exploration of potential reasons for variation in these effects over time in future studies. For example, the attenuation of a protective effect over time in those diagnosed with sensory impairments (supplementary table S6) may be attributable to better clinical awareness of physical health morbidities in people with psychosis.

### Meaning of Findings

We have precisely delineated a substantial incidence of nonorganic psychotic disorder occurring in later life, which our results suggest is distinct from psychosis associated with dementia. There is already evidence that people with VLOSLP have greater preserved functioning compared with those with adult-onset psychosis,^16,17^ but are more socially isolated.^31^ This is consistent with our observations of greater risk with older age, particularly for women,^4,6,35,36^ and given that this population were less likely to have children, or a partner in the 2 years before diagnosis. Together, these findings suggest that this group may harbor unrecognized psychiatric morbidity requiring clinical attention. Our findings also raise questions about the biological and/or social mechanisms underlying increased psychosis risk in older women, which may begin from the well-documented secondary peak in incidence in their late 40s.^32,37^

We found higher rates in later birth cohorts, independent of age, with highest rates in people gestationally exposed to WWII or born thereafter. Although this may reflect greater exposure to malnutrition, or traumatic events occurring during or in the immediate aftermath of the war, we recommend interpreting these results with some caution, give that they could also represent a period effect related to changes in VLOSLP recognition and diagnostic trends occurring from the 1980s.

Psychosis incidence was higher among migrants to Sweden from Africa, North America, Russian-Baltic regions, Europe, and Finland, corresponding with previous VLOSLP findings,^13,14,38^ and those from the younger adult-onset literature.^11,12,39^ Several potential explanations have been proposed, including stressors experienced pre- and post-migration and during migration itself.^11^

Contrary to hypotheses, we found lower rates of VLOSLP in those with hearing and visual impairments. This contrasts several previous small-scale studies.^15,40^ One possibility is that our population-based (rather than clinical) sample reflects under-detection and treatment of sensory impairments in older adults with psychosis at a national-level, as observed for other physical health problems, such as cardiovascular disease, in those with serious mental illness.^41,42^ Such disparities may reflect reduced-help seeking behavior, or provider-level factors, such as the separation of specialist physical and mental health services,^42^ clinical uncertainty in providing suitable care for patients with psychosis, or “diagnostic overshadowing,” where physical symptoms are misattributed to mental illness.^43^

Participants with VLOSLP were more likely to experience a range of social disadvantages than the population at-risk, including lower income, greater social isolation, and adverse life events. One interpretation of these findings is that exposure to structural inequalities and social stressors may have long-lasting effects on psychosis risk into later life. In the younger adult onset literature, low SES has consistently been associated with psychosis risk.^44–46^ Although our findings regarding income could be attributed to social drift during the prodromal phases of psychosis, this interpretation seems less readily applicable to VLOSLP patients, who would have had to maintain sufficient levels of functioning throughout their adult life (ie, survival)^47^ to be at-risk of VLOSLP at cohort entry. Similarly, as suggested for younger adults with psychosis, our findings regarding social isolation could be interpreted causally^48,49^ or may reflect premorbid impairments in social functioning, limiting one’s ability to form and maintain stable intimate relationships; leading to reduced fecundity.^50–52^

Our findings regarding the recent death of a partner, or loss of a child in infancy correspond with previous small-scale studies suggesting that traumatic life events could be associated with VLOSLP,^18–21^ and with the wider epidemiological literature on psychosis in those aged 65 years and younger.^53–55^ Our findings extend this research to suggest that the loss of a child in infancy might convey longstanding—albeit modest—increased risk of psychotic disorder several decades later. On the other hand, this finding may be another manifestation of the association between VLOSLP and long-term social disadvantage. That we did not observe similar effects for the loss of a child at other ages was somewhat surprising; further well-powered studies will be required to understand whether such stressors have more pernicious effects before age 60 years. Further research is now needed to replicate these findings and examine potential biological and psychological mechanisms underlying these associations, with the aim of identifying potential targets for intervention.

## Supplementary Material

Supplementary data are available at *Schizophrenia Bulletin* online.

sby147_suppl_Supplementary_Materials

## Funding

This study was supported by a Sir Henry Dale Fellowship awarded to J.B.K., jointly funded by the Wellcome Trust and the Royal Society (grant no. 101272/Z/13/Z to J.B.K.). J.S. is funded by a Medical Research Council PhD studentship (grant no. MR/K501268/1). J.S., J.B.K., and R.H. are also supported by the National Institute for Health Research, University College London Hospital, Biomedical Research Centre.

## Acknowledgments

We gratefully acknowledge Henrik Dal for his assistance with the preparation of data for this analysis. The authors have declared that there are no conflicts of interest in relation to the subject of this study.
