# Supplementary material for: The Incidence of Nonaffective, Nonorganic Psychotic Disorders in Older People: A Population-based Cohort Study of 3 Million People in Sweden
Source: Schizophr Bull. 2018 Oct 19;45(5):1152–60. doi: 10.1093/schbul/sby147 (PMC6737541; doi:10.1093/schbul/sby147)
Supplement: sby147_suppl_Supplementary_Materials [file sby147_suppl_supplementary_materials.doc]

**Supplementary materials**

Supplementary Table 1: Diagnostic codes
Supplementary Table 2: Missing data (disposable income at age 60)
Supplementary Table 3: Migration sensitivity analysis
Supplementary Table 4: Dementia sensitivity analysis
Supplementary Table 5: Assessment of proportional hazards assumption
Supplementary Table 6: Hazard ratios stratified by time
Supplementary Methods: Further information on estimating exposures

**Supplementary** Table 1- Diagnostic codes

| **Outcome: Non-affective psychotic disorders** | **ICD-10:** F20-F29 |
| --- | --- |
| **Dementia diagnoses** | **ICD-10**: F00, F01, F02, F03 **ICD-9**: 291.2, 292.82, 294.1, 294.2, 294.1x, 294.2x, 331.19, 331.82 |
| **Hearing impairment** | **ICD-10:** H90, H80, Z46.1, Z82.2, Z96.2  **ICD-9:** 369, 387, 95.48, V19.2, V53.2, Z97.4 |
| **Visual impairment** | **ICD-10:** H54, H25, H31.1, H33, H34, H35.3, H35.4, H36, H40, H42, H44.5, Z44.2, Z82.1, Z90.01, Z97.0 **ICD-9:** 389, 360.41, 361, 362.0, 362.3, 362.5, 362.6, 363.4, 365, 366, 368, V19.0, V41.0, V42F, V43.0, V45.78, V52.2 |

**Supplementary Table 2- Missing data (dis**posable income at age 60)

|  | **Missing, N (%)** | **χ^2^ p-value** | |
| --- | --- | --- | --- |
| **Sex:** |  | | P≤.001 |
| Men | 26,782 (1.79) | |  |
| Women | 24,800 (1.64) | |  |
| **Age:** |  | | P≤.001 |
| 60-64 | 11,113 (2.13) | |  |
| 65-69 | 12,906 (1.74) | |  |
| 70-74 | 10,934 (1.89) | |  |
| 75-79 | 9,823 (2.01) | |  |
| 80-84 | 4,666 (1.18) | |  |
| 85-90+ | 2,140 (0.76) | |  |
| **Region of origin:** |  | | P≤.001 |
| Africa | 1,580 (23.75) | |  |
| Asia | 5,369 (23.37) | |  |
| North America | 719 (11.41) | |  |
| Europe | 16,737 (9.39) | |  |
| Sweden | 22,214 (0.84) | |  |
| South America | 733 (8.28) | |  |
| Oceania | 26 (10.32) | |  |
| Other | 152 (65.77) | |  |
| Middle East | 1,302 (11.42) | |  |
| Russia-Baltic | 767 (5.38) | |  |
| Finland | 1,983 (1.69) | |  |
| **Birth period:** |  | | P≤.001 |
| 1920-4 | 3,082 (0.60) | |  |
| 1925-9 | 7,653 (1.73) | |  |
| 1930-4 | 8,791 (2.11) | |  |
| 1934-Aug39 | 8,033 (1.94) | |  |
| WW2-May 1946 | 13,136 (1.71) | |  |
| Post WW2-1949 | 10,887 (2.40) | |  |
| **Child with a psychotic disorder:** |  | | P≤.001 |
| Yes | 51,235 (1.74) | |  |
| No | 347 (0.49) | |  |
| **Death of a child in infancy:** |  | | P≤.001 |
| Had no children | 40,319 (8.42) | |  |
| No children died | 11,159 (0.45) | |  |
| 1 child died | 99 (0.31) | |  |
| 2+ children died | 5 (0.26) | |  |
| **Death of a partner two years before date of exit:** |  | | P≤.001 |
| Had no partner | 38,411 (2.84) | |  |
| Had partner, no partner died | 12,656 (0.8) | |  |
| 1 or more partners died | 515 (0.7) | |  |
| **Visual impairment** |  | | P≤.001 |
| No | 51,390 (2.27) | |  |
| Yes | 192 (0.03) | |  |
| **Hearing impairment** |  | | P≤.001 |
| No | 50,425 (1.78) | |  |
| Yes | 1,157 (0.64) | |  |

**Supplementary Table 3**- Migration sensitivity analysis

|  | **Fully adjusted hazard ratio^a^** | **Sensitivity analysis**^a^**^b^** |
| --- | --- | --- |
| **Offspring with a non-affective psychotic disorder (ref: no offspring with a non-affective psychotic disorder)** | 2.40 (2.23 – 2.58) | 2.40 (2.23 – 2.59) |
| **Region of origin (ref: Sweden)** |  |  |
| Africa | 1.98 (1.44 – 2.72) | 1.54 (1.07 – 2.22) |
| Asia | 1.01 (0.81 – 1.25) | 0.85 (0.67 – 1.08) |
| North America | 1.38 (1.02 – 1.87) | 1.32 (0.97 – 1.80) |
| Europe | 1.32 (1.24 – 1.40) | 1.19 (1.12 – 1.27) |
| South America | 1.11 (0.82 – 1.50) | 0.96 (0.69 – 1.33) |
| Oceania | 1.10 (0.16 – 7.83) | 1.12 (0.16 – 7.96) |
| Middle East | 0.69 (0.49 – 0.96) | 0.57 (0.40 – 0.83) |
| Russia-Baltic | 1.62 (1.37 – 1.91) | 1.54 (1.30 – 1.83) |
| Finland | 1.57 (1.46 – 1.68) | 1.43 (1.33 – 1.54) |
| **Birth period (ref: 1920-1924)** |  |  |
| 1925- 1929 | 1.03 (0.98 – 1.08) | 1.03 (0.98 – 1.08) |
| 1930 - 1934 | 1.34 (1.27 – 1.41) | 1.34 (1.27 – 1.41) |
| 1934 - August 1939 | 1.63 (1.53 – 1.73) | 1.63 (1.54 – 1.74) |
| WW2 - May 1946 | 2.35 (2.20 – 2.50) | 2.31 (2.16 – 2.46) |
| Post-WW2 - 1949 | 3.09 (2.79 – 3.42) | 2.96 (2.66 – 3.29) |
| **Disposable income at age 60 (ref: highest income quartile (4))** |  |  |
| Income quartile 1 (lowest) | 3.07 (2.89 – 3.25) | 3.03 (2.86 – 3.22) |
| Income quartile 2 | 2.72 (2.56 – 2.88) | 2.69 (2.53 – 2.85) |
| Income quartile 3 | 1.46 (1.37 – 1.55) | 1.45 (1.36 – 1.54) |
| **Death of a child (under 12 months of age) (ref: no children died):** |  |  |
| Had no children | 2.41 (2.32 – 2.50) | 2.41 (2.33 – 2.50) |
| 1 or more children died (under 12 months) | 1.20 (1.00 – 1.44) | 1.21 (1.00 – 1.45) |
| 1 or more children died (12 months to 18 years) | 0.99 (0.81 – 1.20) | 1.00 (0.82 – 1.22) |
| **Death of a partner 2 years before date of exit (ref: no partner died)** |  |  |
| Had no partner | 1.86 (1.78 – 1.93) | 1.86 (1.79 – 1.94) |
| 1 or more partners died | 1.14 (1.02 – 1.27) | 1.15 (1.03 – 1.28) |
| **Visual impairment (ref: no visual impairment)** | 0.24 (0.23 – 0.25) | 0.24 (0.23 – 0.25) |
| **Hearing impairment (ref: no hearing impairment)** | 0.55 (0.50 – 0.60) | 0.54 (0.50 – 0.59) |

^a^Adjusted for age, sex, their interaction, and all exposures included in this table
^a^Excluding migrants diagnosed with VLOSLP within two years of arrival to Sweden

**Supplementary Table 4– Dementia sensitivity analysis**

|  | **Fully adjusted hazard ratio^a^** | **Sensitivity analysis^a,b^** |
| --- | --- | --- |
| **Offspring with a non-affective psychotic disorder (ref: no offspring with a non-affective psychotic disorder)** | 2.40 (2.23 – 2.58) | 2.43 (2.26 – 2.62) |
| **Region of origin (ref: Sweden)** |  |  |
| Africa | 1.98 (1.44 – 2.72) | 1.96 (1.42 – 2.71) |
| Asia | 1.01 (0.81 – 1.25) | 1.00 (0.80 – 1.25) |
| North America | 1.38 (1.02 – 1.87) | 1.42 (1.05 – 1.92) |
| Europe | 1.32 (1.24 – 1.40) | 1.31 (1.23 – 1.39) |
| South America | 1.11 (0.82 – 1.50) | 1.08 (0.79 – 1.47) |
| Oceania | 1.10 (0.16 – 7.83) | 1.13 (0.16 – 8.03) |
| Middle East | 0.69 (0.49 – 0.96) | 0.64 (0.45 – 0.91) |
| Russia-Baltic | 1.62 (1.37 – 1.91) | 1.65 (1.39 – 1.95) |
| Finland | 1.57 (1.46 – 1.68) | 1.56 (1.45 – 1.67) |
| **Birth period (ref: 1920-1924)** |  |  |
| 1925- 1929 | 1.03 (0.98 – 1.08) | 1.00 (0.96 – 1.05) |
| 1930 - 1934 | 1.34 (1.27 – 1.41) | 1.29 (1.22 – 1.36) |
| 1934 - August 1939 | 1.63 (1.53 – 1.73) | 1.56 (1.47 – 1.66) |
| WW2 - May 1946 | 2.35 (2.20 – 2.50) | 2.26 (2.11 – 2.41) |
| Post-WW2 - 1949 | 3.09 (2.79 – 3.42) | 2.96 (2.68 – 3.29) |
| **Disposable income at age 60 (ref: highest income quartile (4))** |  |  |
| Income quartile 1 (lowest) | 3.07 (2.89 – 3.25) | 3.13 (2.95 – 3.23) |
| Income quartile 2 | 2.72 (2.56 – 2.88) | 2.75 (2.60 – 2.92) |
| Income quartile 3 | 1.46 (1.37 – 1.55) | 1.46 (1.37 – 1.56) |
| **Death of a child (under 12 months of age) (ref: no children died):** |  |  |
| Had no children | 2.41 (2.32 – 2.50) | 2.42 (2.33 – 2.51) |
| 1 or more children died (under 12 months) | 1.20 (1.00 – 1.44) | 1.17 (0.97 – 1.41) |
| 1 or more children died (12 months to 18 years) | 0.99 (0.81 – 1.20) | 1.01 (0.83 – 1.23) |
| **Death of a partner 2 years before date of exit (ref: no partner died)** |  |  |
| Had no partner | 1.86 (1.78 – 1.93) | 1.87 (1.80 – 1.95) |
| 1 or more partners died | 1.14 (1.02 – 1.27) | 1.11 (0.99 – 1.24) |
| **Visual impairment (ref: no visual impairment)** | 0.24 (0.23 – 0.25) | 0.24 (0.23 – 0.25) |
| **Hearing impairment (ref: no hearing impairment)** | 0.55 (0.50 – 0.60) | 0.53 (0.49 – 0.58) |

^a^Adjusted for all variables in this table, age, sex, region of origin and offspring psychotic disorder
^b^Sensivity analysis excluding those diagnosed with dementia in the two years after diagnosis with VLOSLP (N=421)

**Supplementary Table 5**– Assessment of proportional hazards assumption

| **Variable** | **Schoenfeld residuals test**^a^ |
| --- | --- |
| Offspring with a non-affective psychotic disorder | χ^2^(1)=0.79, P=0.37 |
| Region of origin | χ^2^(10)=12.52, P=0.25 |
| Disposable income at age 60 | **χ^2^(3)=139.70, P≤.001** |
| Birth period | **χ^2^(5)=18.05, P=0.003** |
| Death of a partner | **χ^2^(2)=16.83, P≤.001** |
| Death of a child aged under 12 months | **χ^2^(2)=24.00, P≤.001** |
| Death of a child aged 12 months to 18 years | **χ^2^(2)=24.91, P≤.001** |
| Visual impairment | **χ^2^(1)=347.36, P≤.001** |
| Hearing impairment | **χ^2^(1)=54.79, P≤.001** |

^a^Schoenfeld residuals test, global p-value. Bold denotes possible violation of proportional hazards assumption (see eTable 5)

**Supplementary Table 6– Hazard ratios stratified by time^a^**

| **Variable** | **Time 1^bc^** | **Time 2^bd^** | **Time 3^be^** |
| --- | --- | --- | --- |
| **Disposable income at age 60 (ref: highest income quartile)** |  |  |  |
| Income quartile 1 (lowest) | 4.58 (4.14 – 5.07) | 3.01 (2.72 – 3.32) | 2.01 (1.82 – 2.22) |
| Income quartile 2 | 4.03 (3.65 – 4.46) | 2.44 (2.21 – 2.69) | 1.92 (1.74 – 2.12) |
| Income quartile 3 | 1.68 (1.51 – 1.87) | 1.34 (1.22 – 1.51) | 1.30 (1.17 – 1.44) |
| **Death of a partner** |  |  |  |
| Had no partner | 1.93 (1.81 – 2.05) | 1.84 (1.72 – 1.96) | 1.80 (1.67 – 1.94) |
| 1 or more partners died | 0.90 (0.71 – 1.15) | 1.00 (0.83 – 1.21) | 1.36 (1.16 – 1.60) |
| **Death of a child (ref: had children, none died)** |  |  |  |
| Had no children | 2.43 (2.28 – 2.59) | 2.31 (2.16 – 2.46) | 2.44 (2.29 – 2.59) |
| 1 or more children died aged under 12 months | 1.04 (0.79 – 1.38) | 1.45 (1.09 – 1.94) | 1.18 (0.76 – 1.83) |
| 1 or more children died aged 12 months – 18 years | 0.78 (0.53 – 1.13) | 1.07 (0.78 – 1.47) | 1.11 (0.80 – 1.54) |
| **Birth period (ref: 1920-1924)** |  |  |  |
| 1925 - 1929 | 0.85 (0.76 – 0.95) | 1.20 (1.11 – 1.30) | 0.89 (0.83 – 0.95) |
| 1930 - 1934 | 1.26 (1.12 – 1.41) | 1.34 (1.34 – 1.59) | 1.23 (1.11 – 1.36) |
| 1934 - August 1939 | 1.69 (1.52 – 1.89) | 1.65 (1.50 – 1.81) | 2.40 (0.59 – 9.74) |
| Sep 1939 - May 1946 (gestational exposure to WWII) | 2.48 (2.25 – 2.73) | 1.84 (1.58 – 2.10) | - |
| Jun 1946 - 1949 | 3.16 (2.79 – 3.57) | - | - |
| **Visual impairment (ref: no visual impairment)** | 0.14 (0.12 – 0.15) | 0.17 (0.15 – 0.19) | 0.34 (0.32 – 0.36) |
| **Hearing impairment (ref: no hearing impairment)** | 0.34 (0.28 – 0.40) | 0.45 (0.39 – 0.56) | 0.70 (0.62 – 0.80) |

^a^Adjusted for all variables in this table, age, sex, region of origin and offspring psychotic disorder
^b^Time split into centiles based on failure rates
^c^Time 1: January 1980 – May 1988 ^d^Time 2: May 1988 – October 1996 ^e^Time 3: October 1996 – December 2011

**Supplementary Methods**

*Further information on estimating gestational exposure to WWII*

To investigate the possible role of gestational exposure to maternal stressors experienced during World War II (WWII: 1^st^ Sep 1939-2^nd^ Sep 1945), we assumed a typical gestation of 40 weeks (280 days). All participants born from the first day of WWII (1^st^ Sep 1939), up until to 279 days after the end of the war (2^nd^ Sep 1945 + 279 days = 8^th^ June 1946) were classified as having had some gestational exposure to WWII. Date of birth in the Register of the Total Population is only available for research purposes for the month and year of birth, with all participants given a birthday of the 15^th^ of their birth month. For this reason, our gestational exposure definition excluded all people born in June 1946, making our gestational exposure window effectively Sep 1945 – May 1946. Remaining participants were coded to the following birth periods, based on their date of birth: 1920-1924, 1925-1929, 1930-1933, 1934-Aug 1939, June 1946-1949. Although Sweden remained neutral during WWII, it nevertheless remained subject to naval blockades, food and fuel shortages, rationing (until 1951), accidental bombings and threats of invasion throughout this period.

*Further information on estimating death of a partner*

Two assumptions were made to allow us to define partner deaths in the two years prior to cohort exit. First, prior to the LISA, information on partner statuses were only available from quinquennial censuses (i.e. 1985, 1980, 1975). We therefore assumed that for participants who exited the cohort prior to 1990, their partner status was consistent with their last census entry (i.e. someone who left the cohort between 1986-1989 would be coded to their partner status in the 1985 census). Second, the exact date at which partner status was recorded (via Census or the LISA) was not given, therefore partner status recorded in a given year was assumed to apply for the whole year.
